# Epigenetic Connections in Malocclusion

**DOI:** 10.3390/ijms27146380

**Published:** 2026-07-17

**Authors:** Elzbieta Pawlowska, Maria Mitus-Kenig, Janusz Blasiak

**Affiliations:** 1 Department of Pediatric Dentistry, Medical University of Lodz, 92-213 Lodz, Poland; 2 Department of Periodontology, Preventive Dentistry and Oral Pathology, Faculty of Medicine, Jagiellonian University, 31-532 Krakow, Poland; maria.mitus-kenig@uj.edu.pl; 3 Faculty of Medicine, Mazovian University in Plock, 09-402 Plock, Poland

**Keywords:** malocclusion, epigenetic, DNA methylation, histone modifications, non-coding RNAs, RNA chemical modifications, orthodontic forces, orthodontic tooth movement

## Abstract

Malocclusion arises from complex interactions among genetic, environmental, and developmental factors. While genetic contributions are well established, epigenetic mechanisms, including DNA methylation, histone modifications, non-coding RNAs, and RNA chemical modifications, have emerged as plausible regulators of craniofacial growth and dentoalveolar remodeling. This structured narrative review critically evaluates current evidence on the role of epigenetic regulation in the development of malocclusion and in orthodontic tooth movement. Most available data derive from in vitro studies, animal models, and investigations of related craniofacial processes rather than from direct analyses of defined malocclusion phenotypes in humans. Consequently, the evidence base is largely indirect and heterogeneous. To address this limitation, we applied a qualitative appraisal framework that considered study design, methodological rigor, and the directness of the evidence. Experimental findings indicate that epigenetic mechanisms are dynamically regulated by mechanical forces and may influence osteogenesis, chondrogenesis, periodontal remodeling, and individual variability in orthodontic response. However, robust causal studies directly linking specific epigenetic modifications to malocclusion phenotypes remain lacking. Although biological plausibility is strong, a substantial gap persists between mechanistic insights and clinical translation. At present, the clinical utility of epigenetic markers in orthodontic diagnosis, treatment strategy, or prognosis remains limited. Future research should prioritize well-designed longitudinal human studies integrating epigenetic profiling with clearly defined malocclusion phenotypes to establish causal relationships and enable clinically relevant applications.

## 1. Introduction

Malocclusion is a complex trait characterized by the interplay of genetic, environmental, and developmental factors [1]. Although heritability studies have demonstrated a substantial genetic contribution to craniofacial morphology, they do not fully account for the considerable phenotypic variability observed among individuals with similar genetic backgrounds. This discrepancy underscores the importance of regulatory mechanisms that mediate gene–environment interactions during craniofacial development. Epigenetics provides a biologically plausible framework for such regulation [2]. Epigenetic mechanisms, including DNA methylation, histone modifications, chromatin remodeling, non-coding RNA activity, and RNA modifications, govern gene expression without altering the underlying DNA sequence and play essential roles in osteogenesis, chondrogenesis, and odontogenesis [3,4,5,6]. Through these processes, epigenetic regulation may influence craniofacial growth, skeletal patterning, and the adaptive response of dentoalveolar tissues to environmental stimuli, including mechanical loading, nutrition, airway function, and systemic factors [7].

Increasing evidence indicates that epigenetic regulation is dynamic and responsive to external cues, including mechanical forces [8]. In orthodontics, epigenetic changes have been observed in periodontal ligament cells and bone tissues under controlled loading, suggesting their role in tissue remodeling and orthodontic tooth movement (OTM) [9]. These findings support the concept that epigenetic mechanisms may contribute not only to normal craniofacial development but also to variability in treatment response and outcomes [10].

Despite strong biological plausibility, the current body of evidence is marked by significant limitations. Most available data come from in vitro experiments, animal models, or studies of general craniofacial biology rather than from investigations directly examining defined malocclusion phenotypes in humans [11]. Consequently, most findings remain indirect, and robust causal evidence linking specific epigenetic modifications to specific types of malocclusion is still lacking. Moreover, a substantial gap persists between mechanistic insights and their translation into clinical orthodontic practice.

Although the current evidence base is limited and largely indirect, synthesizing these data is essential at this stage of the field to prevent overinterpretation, define the boundaries of current knowledge, and establish priorities for rigorous future research. The present work therefore aims to evaluate the evidence on epigenetic mechanisms in malocclusion and orthodontic biology, with particular attention to the strength, directness, and translational relevance of available studies. By integrating current findings with a critical appraisal framework, this review seeks to clarify the role of epigenetics in dentofacial development, identify key knowledge gaps, and outline priorities for future research toward clinically meaningful applications. To enhance transparency and address these limitations, a structured approach to evidence appraisal was used, as detailed in the following section.

## 2. Evidence Assessment Strategy

### 2.1. Study Design and Literature Search

This work was conducted as a structured narrative and perspective review focusing on the role of epigenetic mechanisms in the development and treatment of malocclusion. Given the heterogeneity of available studies and the predominance of preclinical and mechanistic evidence, a formal systematic review was not feasible. Instead, a structured qualitative approach incorporating elements of critical appraisal was adopted to improve the transparency and interpretability of the evidence base.

A comprehensive literature search was performed using the following databases: PubMed, Embase, Scopus, Google Scholar, ScienceDirect, and the Cochrane Library. The search covered primarily the last 10 years (2015–2025), although earlier foundational studies were included where relevant. Search terms were combined using Boolean operators and included: “malocclusion” AND “epigenetic”; “DNA methylation” AND “craniofacial”; “histone modification” AND “bone remodeling” (“miRNA” OR “non-coding RNA”) AND “orthodontic tooth movement”; “epitranscriptomics” AND “craniofacial development”. No language restrictions were applied. All types of publications were considered, including original research (human, animal, and in vitro studies), systematic reviews, narrative reviews, and meta-analyses.

### 2.2. Study Selection, Categorization, Directness Assessment, and Eligibility Criteria

Studies were included if they met at least one of the following criteria: (1) investigated epigenetic mechanisms (DNA methylation, histone modifications, non-coding RNAs, or RNA modifications); (2) examined craniofacial development, bone remodeling, or orthodontic tooth movement; and (3) explored relationships potentially relevant to malocclusion etiology or treatment. Studies were excluded if they: (1) did not involve epigenetic mechanisms; (2) were unrelated to craniofacial or dental biology; and (3) lacked mechanistic or biological relevance. Given the exploratory nature of the field, studies were not excluded solely on the basis of design; instead, their methodological quality and relevance were evaluated qualitatively.

To address the variability and largely indirect nature of the available evidence, studies were stratified by relevance to malocclusion: Level I (direct evidence): human studies directly linking epigenetic changes to defined malocclusion phenotypes; Level II (translational evidence): animal studies investigating craniofacial growth or orthodontic models; Level III (mechanistic in vitro evidence): studies of PDL cells, osteoblasts, or stem cells under controlled conditions; Level IV (indirect biological evidence): studies of general craniofacial development, bone biology, or epigenetic regulation not specific to malocclusion; and Level V (conceptual/hypothesis-generating evidence): theoretical frameworks and extrapolations. Additionally, each study was evaluated for directness, defined as the extent to which the investigated epigenetic mechanism was linked to clinically observable malocclusion outcomes rather than broader craniofacial or cellular processes.

### 2.3. Quality Assessment and Risk of Bias

Because many studies were preclinical or heterogeneous in design, formal standardized tools were adapted rather than applied rigidly. The following appraisal principles were used: (1) animal studies: assessed in accordance with the ARRIVE (Animal Research: Reporting of In Vivo Experiments) guidelines, with a focus on experimental control, reproducibility, and reporting transparency; (2) in vitro studies: evaluated using domains inspired by OHAT (Office of Health Assessment and Translation), including exposure characterization, reproducibility, and outcome measurement; and (3) human studies: assessed using criteria analogous to the Newcastle–Ottawa Scale (NOS), with a focus on study design, comparability, and outcome validity.

Across all studies, the following domains were considered: study design and methodological rigor, sample size and reproducibility, clarity of epigenetic measurements, biological plausibility, risk of confounding, and strength and consistency of associations. For review articles, methodological quality was assessed qualitatively using AMSTAR-2 (A MeaSurement Tool to Assess systematic Reviews 2) principles, with particular attention to the comprehensiveness of the search strategy and the risk-of-bias evaluation. Across all study types, the following domains were systematically considered: study design and sample characteristics, methodological transparency, reproducibility, clarity of epigenetic measurements, and the extent to which findings could be directly linked to clinically defined malocclusion phenotypes. Studies were not excluded based on quality assessment; instead, their strengths and limitations were explicitly incorporated into the narrative synthesis, with particular attention to the directness and translational relevance of the evidence.

### 2.4. Data Synthesis and Limitations

Due to heterogeneity in study design, populations, and experimental models, a quantitative meta-analysis was not performed. Instead, findings were synthesized descriptively, with emphasis on consistency across studies, the level of evidence and directness, mechanistic plausibility, and translational relevance. Where appropriate, mechanistic findings from animal and in vitro models were interpreted in the context of their applicability to human malocclusion.

This review is subject to limitations inherent in narrative synthesis, including potential selection bias and the absence of quantitative effect estimation. Furthermore, the predominance of indirect and preclinical evidence restricts causal inference and limits the ability to draw definitive conclusions about epigenetic mechanisms in clinical malocclusion.

## 3. Epigenetics and Its Role in Dentistry

Before discussing specific epigenetic mechanisms, it is important to distinguish among three related but distinct concepts: epigenetic regulation of craniofacial development, epigenetic regulation of orthodontic tissue remodeling, and epigenetic contributions to malocclusion etiology. Although substantial evidence supports epigenetic involvement in craniofacial development and OTM, direct evidence linking specific epigenetic alterations to defined malocclusion phenotypes remains limited. Consequently, many mechanistic links discussed below should be viewed as biologically plausible hypotheses rather than established causal pathways.

The three primary components of epigenetic regulation of gene expression are DNA methylation, post-translational histone modification, and non-coding RNA activity [12]. In recent years, attention has also shifted to RNA modifications that do not alter RNA sequence but influence gene expression [13]. Thus, epigenetics may be considered alongside epitranscriptomics, and the epigenome alongside the epitranscriptome.

### 3.1. DNA Methylation

**Figure 1:**
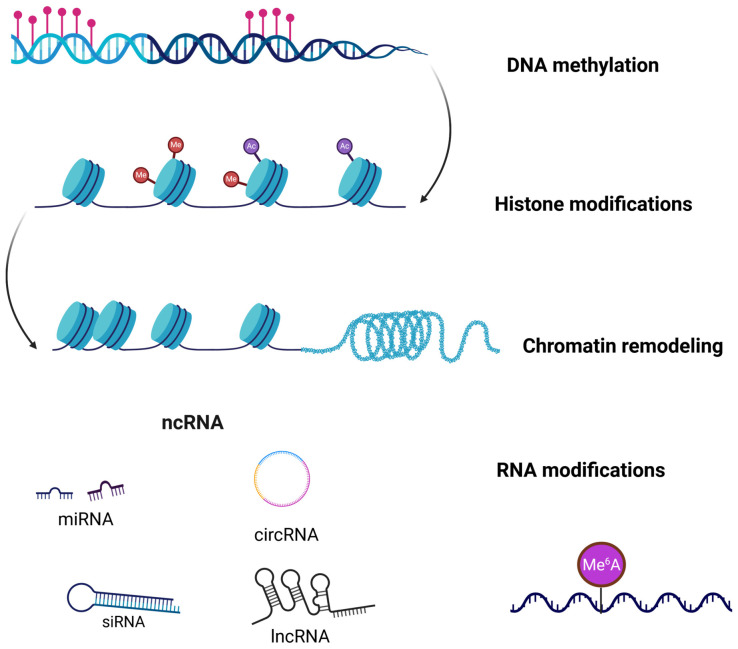
The main epigenetic modifications of the genome include DNA methylation (red dots) and post-translational histone modifications, here represented by methylation (Me) and acetylation (Ac). DNA methylation is recognized by methylation-specific proteins that alter the expression of methylated genes, while histone modifications recruit proteins that remodel chromatin, thereby altering the accessibility of transcriptional factors to genes and modulating their expression, which could be further changed by non-coding RNAs (ncRNAs), represented here by micro-RNA (miRNA), circular RNA (circRNA), short-interfering RNA (siRNA), and long non-coding RNA (lncRNA). RNA, resulting from transcription, can also be modified by the addition of a chemical group, here represented by N6-methyladenosine (Me^6^A). Created in BioRender. Michlewska, S. (2026) https://BioRender.com/36ppoor. (accessed on 7 July 2026).

DNA methylation is the most extensively studied epigenetic modification and involves the addition of a methyl group (CH_3_) from S-adenosylmethionine to cytosine or adenine in DNA by DNA methyltransferase, resulting in the formation of 5-methylcytosine (5-metC, Figure 1) [14]. Humans possess three DNA methyltransferases: DNMT1, DNMT3A, and DNMT3B, as well as DNMT3L, which lacks catalytic activity but assists DNMT3A/B. Another methyltransferase, DNMT2, methylates cytosine in both DNA and RNA.

In humans, DNA methylation occurs almost exclusively within the dinucleotide 5′-CpG-3′, and the human genome contains regions with a significantly higher frequency of these dinucleotides than average; these regions are called CpG islands [15]. They are often associated with gene regulatory elements such as promoters and enhancers and are typically unmethylated under normal conditions. Methylated DNA can be specifically recognized by proteins known as methyl-CpG-binding proteins, which can recruit other proteins that block transcription factor binding, thereby inhibiting gene expression; however, this is only one of several mechanisms by which DNA methylation affects gene expression.

Methylated DNA undergoes passive demethylation, primarily due to the lack of DNMT1 activity during replication and the “dilution” of methylation with each cell division [16]. In addition to passive demethylation, active DNA demethylation also occurs and involves enzymes of the TET (Ten-Eleven Translocation) family and thymine DNA glycosylase [17].

### 3.2. Post-Translational Covalent Histone Modifications

The relationship between the length of human nuclear DNA (about 2 m) and the average nuclear diameter in human cells (around 6 micrometers) requires the genome to be packaged in a compact yet highly organized way—not only to “fit” DNA into the nucleus but also to allow proper reading of genetic information. This organization is achieved by forming complexes of DNA with proteins, classified as histones and non-histone proteins; the ordered, dynamic complex is called chromatin. Histones undergo post-translational covalent modifications, primarily in their N-terminal tails, which extend from histone complexes and are accessible to chromatin-modifying enzymes [18]. Histone modifications include methylation, acetylation, phosphorylation, ubiquitination, and others, produced by histone-modifying enzymes that form large protein complexes with DNA-binding proteins and chromatin-remodeling enzymes (Figure 1). These complexes are involved in the epigenetic regulation of DNA replication, transcription, DNA repair, and other cellular processes independently of DNA sequence.

Histone modifications mainly affect the expression of genes whose promoters are in chromatin regions containing these modifications [19]. The pattern of histone modifications is interpreted by specific proteins, which then interact with additional chromatin-remodeling proteins to regulate chromatin accessibility for proteins that directly control gene expression. Generally, enzymes involved in establishing, maintaining, and modifying the histone code are classified into three groups: writers—enzymes that add modifications; erasers—enzymes that remove modifications; and readers—proteins that recognize modifications.

In certain chromatin regions, DNA methylation patterns align with post-translational histone modifications [20]. In areas where DNA methylation promotes gene silencing, histone tails undergo modifications that prefer chromatin condensation, making it more difficult for transcription factors to access DNA. This synergy between histone modifications and DNA methylation can enhance the potential for gene silencing or activation.

### 3.3. Non-Coding RNAs

Coding RNA makes up only about 4% of the human transcriptome, while the remaining 96% consists of non-coding RNA (ncRNA), which can be categorized into constitutive ncRNA involved in basic metabolism, such as ribosomal RNA (rRNA) and transfer RNA (tRNA), and regulatory ncRNA (Figure 1). The latter group is divided into short non-coding RNA (snRNA, less than 200 nt) and long non-coding RNA (lncRNA, more than 200 nt) [21]. Within these groups, several subclasses exist, with microRNA (miRNA) being the most extensively studied. Various mechanisms determine ncRNA’s role in gene expression regulation, mainly through interactions with mRNA. Some ncRNAs interact with other ncRNAs, creating a sponging effect, as a single ncRNA may contain binding sites for many other ncRNAs, preventing them from interacting with their target mRNAs.

MicroRNAs regulate gene expression through RNA interference (RNAi), where miRNA recognizes a complementary region in target mRNA, forming a double-stranded RNA structure that is recognized and degraded by protein complexes containing nucleases [22]. MicroRNAs are found in body fluids and act as easily accessible biomarkers for certain diseases. Small interfering RNAs (siRNAs) are short double-stranded RNA fragments that, through the RNA interference mechanism, specifically silence gene expression by degrading complementary mRNA [23]. Another type of non-coding RNA is circular RNA (circRNA), which, because of its closed structure and lack of free ends, is more stable than linear RNA and less susceptible to exonuclease activity. Its circular shape and size (>100 nt) make circRNAs suitable for binding or sequestering other ncRNAs, including miRNAs [24]. In addition to this sponging effect, circRNAs may also regulate gene expression through other mechanisms, including direct interaction with the promoter–RNA polymerase II complex and binding to regulatory proteins. Some circRNAs can even undergo translation into functional proteins.

In cells, various RNA molecules, including mRNA, lncRNA, circRNA, and pseudogenes, can contain miRNA response elements (MRE). These RNA species can compete with target mRNA for miRNA binding, effectively “bypassing” the miRNA and decreasing its ability to regulate target transcripts. This process is known as endogenous competition, and RNAs with MRE sequences are called competitive endogenous RNAs (ceRNAs) [25]. Endogenous competition primarily affects gene expression by stabilizing mRNA levels of genes that must remain within a specific range at a given time.

PIWI-interacting RNA (piRNA) is another ncRNA involved in regulating gene expression. However, there is currently no documented link between piRNA biology and malocclusion.

### 3.4. RNA Modifications

In addition to epigenetic modifications of the genome, one can also consider modifications of the transcriptome—chemical changes to RNA that influence gene expression and other cellular functions [26]. These modifications can happen in all types of RNA, including mRNA, tRNA, rRNA, and ncRNA. Major RNA modifications include N6-methyladenosine (m6A, Figure 1), which affects mRNA stability and translation; Ψ (pseudouridine), which alters RNA structure and function; and m5C (5-methylcytosine), which is involved in translation regulation. As with histone modifications, the establishment, maintenance, and remodeling of the epitranscriptome involve three groups of enzymes: writers, erasers, and readers.

### 3.5. Significance of Epigenetics in Craniofacial and Dental Development and Pathology

Epigenetic mechanisms are essential in various areas of dentistry by regulating how oral tissues develop, maintain homeostasis, respond to environmental factors, and undergo disease-related changes [27,28]. Unlike traditional genetics, epigenetic regulation offers a flexible and reversible system that allows cells to adjust gene expression in response to biochemical, mechanical, microbial, and systemic signals throughout life.

In craniofacial and dental development, epigenetic programs direct neural crest cell differentiation, tooth formation, root development, and alveolar bone growth [29]. Patterns of DNA methylation and histone modification control the expression of key developmental genes, including msh homeobox 1 (MSX1), paired box 9 (PAX9), runt-related transcription factor 2 (RUNX2), distal-less homeobox (DLX) family members, and bone morphogenetic protein (BMP) and fibroblast growth factor (FGF) pathway genes, ensuring the proper formation of dental tissues, periodontal structures, and maxillofacial bones [30,31].

In periodontology, epigenetic changes influence inflammatory responses, osteoclastogenesis, and tissue destruction in periodontal disease [32]. Hypermethylation or hypomethylation at loci linked to cytokine signaling by interleukins IL6 and IL1B, extracellular matrix (ECM) remodeling by matrix metalloproteinases (MMPs), and bone metabolism (receptor activator of nuclear factor κB ligand/osteoprotegerin (RANKL/OPG)) affects disease susceptibility, progression, and treatment response [32,33,34]. In endodontics, epigenetic regulation affects pulpal inflammation, odontoblast differentiation, and reparative dentinogenesis [35]. Non-coding RNAs and histone acetylation patterns are key players in the regenerative response after injury or infection, providing potential targets for biologically enhanced regeneration [36].

In oral pathology, epigenetic dysregulation plays a key role in the initiation and progression of oral potentially malignant disorders, including oral squamous cell carcinoma [37,38]. Aberrant promoter methylation of tumor suppressor genes, extensive chromatin remodeling, and altered miRNA expression profiles contribute to oncogenesis and can serve as diagnostic, prognostic, or therapeutic biomarkers [39].

In implant dentistry and bone regeneration, epigenetic pathways govern osteoblast maturation, osseointegration, and bone healing [40,41]. Epigenetic modifiers, such as histone deacetylase (HDAC) inhibitors or miRNA-based therapeutics, are being studied to enhance bone regeneration and increase implant stability [42,43].

While these mechanistic observations support the biological plausibility of an epigenetic contribution to skeletal malocclusion, they should not be interpreted as evidence that altered DNA methylation directly causes Class II or Class III phenotypes in humans. At present, these relationships remain largely hypothetical and require confirmation in well-characterized human cohorts.

**Figure 2:**
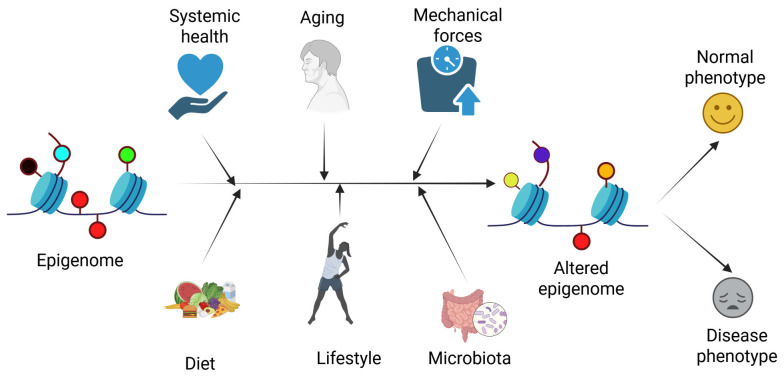
The epigenome, defined as the genome together with its complete set of epigenetic modifications, can be altered by a variety of factors, including systemic health, aging, mechanical stimuli, diet, lifestyle, and microbiota composition. Such epigenomic changes, represented here by different colors, may manifest in either normal physiological variation or in the development of disease phenotypes. Created in BioRender, Michlewska, S (2026) https://BioRender.com/u5nxw2p (accessed on 2 July 2026).

Collectively, epigenetics offers a unifying framework that explains how genetic predisposition interacts with environmental influences, including mechanical forces, microbiota, systemic health, aging, and lifestyle factors, to shape oral health and disease (Figure 2). As epigenetic insights deepen, they may be helpful for more accurate diagnostics, personalized treatment strategies, and biologically targeted therapies in dental medicine.

## 4. Epigenetics of Malocclusion

The results presented in the previous section, along with the consideration of epigenetic phenomena in dentistry-related effects, justify their inclusion in malocclusion. It must be stressed that experimental evidence is weak, but we want to provide arguments that this subject is worth investigating [17].

### 4.1. DNA Methylation

DNA methylation is one of the most often studied epigenetic mechanisms in craniofacial biology. During craniofacial development, DNA methylation patterns regulate neural crest cell specification, migration, chondrogenesis, osteogenesis, and tooth morphogenesis [4,44].

Bone development genes, such as *MSX1, PAX9, RUNX2*, and fibroblast growth factor (FGFR2), are strongly associated with craniofacial disorders and tooth agenesis through genetic mutations, transcriptional regulation, and signaling pathway dysfunction [30,45,46]. Although these findings primarily derive from syndromic or developmental studies rather than from malocclusion itself, they highlight methylation-sensitive pathways that also affect skeletal class patterns and dental arch shape.

Bone remodeling and orthodontic tooth movement are essential elements of orthodontic therapy, particularly in the use of orthodontic appliances to correct malocclusions. Periodontal ligament (PDL) cells subjected to compressive and tensile forces exhibit dynamic changes in DNA methylation that affect genes involved in osteoclastogenesis (RANKL, OPG), osteogenesis (BMP2, ALPL), and inflammatory mediators [47,48]. This supports the concept that DNA methylation modulates individual variability in orthodontic tooth movement and tissue adaptation.

The following examples primarily derive from developmental biology and craniofacial growth studies and therefore provide indirect rather than direct evidence for the involvement of DNA methylation in the pathogenesis of malocclusion. Several studies on DNA methylation show potential relevance to malocclusion. Variability in DNA methylation of craniofacial growth regulators may contribute to skeletal Class II or III patterns. Although no study directly links variability in DNA methylation patterns to class II/III malocclusion, some craniofacial development papers show that DNA methylation is a major regulator of cranial neural crest cells (CNCCs) [44,49,50]. Skeletal Class II and III patterns result from disproportionate growth of the maxilla and mandible. Cranial neural crest cells (CNCCs) guide the development of the maxilla, mandible, and midface. If the epigenetic regulation of CNCCs’ proliferation, migration, or differentiation is altered, the resulting skeletal structures may deviate from normal growth patterns. DNA methylation is crucial for proper CNCC function; disruptions can affect the development of craniofacial skeletal elements. Therefore, variable DNA methylation in growth-regulating genes, including homeobox genes, FGFs, and BMP/transforming growth factor (TGF) pathways, could influence mandibular or maxillary growth toward a class II or III pattern [43]. Craniofacial morphogenesis heavily relies on epigenetic regulators, and variations in these regulators contribute to differences in facial structure among individuals. This directly supports the idea that epigenetic differences may predispose people to specific types of malocclusion [51]. Epigenetic patterns in CNCCs influence facial shape. Even minor methylation differences can lead to varied skeletal outcomes, affecting jaw length, mandibular rotation, or sagittal discrepancies observed in class II/III patients [52]. Epigenetic variability may help explain why (1) some class II/III patterns occur without strong Mendelian inheritance; (2) siblings can display significantly different jaw growth patterns; (3) environmental factors such as diet, posture, and endocrine conditions seem to influence craniofacial growth more than genetics alone; and (4) methylation signatures may indicate environmental exposures like mouth breathing, diet consistency, and endocrine factors.

Epigenetic drift, which refers to random age-related changes in DNA methylation, could affect when malocclusion appears or how severe it is by impacting tissues that control craniofacial growth and dental support [53,54]. DNA methylation changes gradually with age and serves as a reliable marker of biological aging. These modifications can influence gene expression in bone, cartilage, and periodontal tissues [55]. Changes in DNA methylation patterns accumulate predictably and are linked to biological aging pathways [55]. This can be clinically manifested by (1) late-onset mandibular growth changes, such as late mandibular forward rotation, which may be partly driven by age-related epigenetic drift; (2) increased crowding in adults, potentially reflecting a decline in PDL integrity and bone remodeling capacity linked to methylation drift; and (3) variability in malocclusion progression, even among adults with similar initial conditions, possibly due to differences in epigenetic aging rates. Therefore, epigenetic drift might help explain the unpredictable worsening of Class III or late mandibular growth spurts.

Differential DNA methylation may aid in predicting responsiveness to functional appliances in growing patients [49]. Functional appliances depend on the patient’s biological capacity to modify jaw growth, especially the mandible. If DNA methylation patterns influence mechanosensitivity or growth responsiveness, they could predict treatment outcomes. Mechanical forces applied to PDL cells cause measurable DNA-methylation changes, such as increased methylation of RANKL, and affect the expression of osteogenic and remodeling genes. This demonstrates that craniofacial tissues experience force-dependent epigenetic remodeling [42]. Mechanotransduction is a fundamental driver of craniofacial adaptation, and epigenetic regulators influence this pathway. This provides a mechanistic basis for predicting which patients have high versus low remodeling potential [51]. Clinical implications of these effects may include (1) identifying “high-responders” with strong osteogenic responsiveness to mandibular advancement appliances; (2) flagging low-responders whose tissues exhibit impaired mechanotransduction or slow remodeling; and (3) enabling precision orthodontics by selecting appropriate timing for growth-modification therapy.

### 4.2. Histone Modifications

Histone modifications, including acetylation, methylation, phosphorylation, ubiquitination, and sumoylation, regulate DNA accessibility and chromatin architecture. These marks determine whether gene loci involved in craniofacial morphogenesis are actively transcribed or silenced.

Studies in experimental models demonstrate that histone acetyltransferases (HATs) and HDACs regulate the expression of genes required for osteoblast and chondrocyte differentiation, including collagen type I alpha 1 chain (COL1A1), SOX9, and RUNX2 [44]. HDAC inhibitors have been shown to promote osteogenic differentiation, indicating epigenetic regulation of skeletal growth potential [56]. Class II and III malocclusions result from mismatched jaw growth, such as mandibular deficiency, mandibular prognathism, and maxillary overgrowth. If HDAC/HAT activity changes the expression of RUNX2 (which is involved in osteoblast differentiation) or SOX9 (which is involved in chondrogenesis in the mandibular condyle), this can alter the growth direction and rate of the mandible. Therefore, hypoacetylation or increased HDAC activity could suppress mandibular condylar chondrogenesis, leading to mandibular retrognathia (Class II). Conversely, hyperacetylation or HDAC inhibition could promote bone deposition or condylar growth, potentially affecting prognathism or causing asymmetry (Class III). It is important to note that the following discussion is based primarily on experimental and developmental studies rather than on direct investigations of patients with defined malocclusion phenotypes. Consequently, the proposed links between histone modifications and skeletal malocclusion should be interpreted as mechanistically informed hypotheses.

Among histone deacetylases, Class IIa HDACs, particularly HDAC4, are the most plausible epigenetic regulators of mandibular condylar growth. HDAC4 integrates mechanical and developmental cues in cartilage by repressing RUNX2- and MEF2-dependent chondrocyte hypertrophy and endochondral ossification [57]. Increased HDAC4 activity or global hypoacetylation may therefore suppress condylar chondrogenesis and longitudinal mandibular growth, contributing to mandibular retrognathia characteristic of Class II patterns. Conversely, reduced HDAC4 activity or pharmacological HDAC inhibition may promote hypertrophy and ossification, facilitating condylar overgrowth or asymmetric mandibular development consistent with Class III phenotypes [58]. In an animal study investigating the role of HDCA1-11 in aging in mandibular condylar cartilage, it was observed that HDCA1, 2, 3, 4, and 8 showed a time-dependent pattern of expression in the temporomandibular joint condyle [59]. Alterations in the growth and adaptive remodeling of the mandibular condyle can contribute to the development of class II malocclusion [60]. However, direct evidence demonstrating that altered HDAC4 activity causes Class II or Class III malocclusion in humans is currently lacking. The proposed relationships represent theoretical models derived from developmental and mechanistic observations and remain to be validated in clinical studies.

In PDL fibroblasts, mechanical stimulation modifies H3 acetylation and H3K27 trimethylation, influencing pathways crucial for osteoclast recruitment and remodeling [61]. H3 acetylation is a recognized marker of open chromatin that promotes the transcription of remodeling- and osteoclast-related genes. Increased H3 acetylation under compression supports the idea that mechanical loading may alter epigenetic states in PDL cells, thereby affecting osteoclast recruitment and local remodeling, both of which are essential for orthodontic tooth movement and malocclusion biomechanics. PDL fibroblasts show global H3K27 acetylation changes that regulate osteogenic and remodeling pathways [62,63]. Although not force-specific, this study indicates that H3K27-related histone modifications are key regulators of osteogenic gene expression in PDL fibroblasts. Since compressive and tensile forces attract osteoclasts to the compression side, the discovery that H3K27ac governs bone remodeling genes supports a mechanistic link between mechanical stress, histone modification, and osteoclast-mediated bone resorption. These findings indirectly suggest a role for histone modifications in the biomechanics involved in malocclusion development.

In summary, differences in craniofacial growth rates among individuals may be partly attributable to variation in histone modification patterns within growth centers such as the condylar cartilage and the midpalatal suture. Histone marks could mediate tissue responses to orthopedic or orthodontic forces, thereby affecting skeletal adaptation. Environmental factors, such as hypoxia due to upper airway issues, endocrine disruptors, and dietary consistency, might influence histone acetylation patterns associated with occlusal development.

### 4.3. Non-Coding RNAs

Non-coding RNAs add a complex regulatory layer by modulating mRNA stability, translation, or chromatin state. They are tightly integrated with mechanical force responses, craniofacial growth, and bone turnover.

MiRNAs such as miR 29, miR 21, miR 146a, and miR 34 family members regulate osteoblast and osteoclast differentiation. In particular, miR-29a/b/c promote osteoclast commitment, differentiation, and migration, increasing TRAP^+^ osteoclast formation [53]. miR-21 was shown to play a dual role, promoting osteogenesis and regulating osteoclast differentiation (via PDCD4, RANKL/OPG, and FasL pathways) [63,64].

Mechanical strain in PDL cells alters miRNA expression patterns that, in turn, regulate RANKL/OPG, MMPs, ITGB1, and other remodeling genes. Studies on human PDL fibroblasts exposed to tensile or compressive forces show significant upregulation of miR-21 and miR-146a, with mechanical force-dependent changes in chromatin state and gene expression, including RANKL and OPG [51,65]. miR-146a regulates osteogenesis, inhibits osteoclastogenesis, and modulates inflammation through the tumor necrosis factor receptor-associated factor 6 (TRAF6/NF-κB) pathways [66]. miR-146a knockout mice exhibit increased osteoclast activity and abnormal bone turnover, confirming its regulatory role [67]. In osteoblast-osteoclast coculture, inhibiting miR-21 decreases the expression of MMP-9, a crucial matrix metalloproteinase involved in bone remodeling [68].

Malocclusion can worsen or correct depending on the rates of bone resorption and formation. lncRNA Nron is one of the few lncRNAs with direct, experimentally demonstrated effects on osteoclastogenesis during orthodontic tooth movement, making it highly relevant to malocclusion pathogenesis [69]. That study shows that lncRNA Nron regulates osteoclast maturation by controlling the nuclear translocation of nuclear factor of activated T-cells, cytoplasmic 1 (NFATc1). Overexpression of lncRNA Nron reduces osteoclast number and bone resorption, slowing orthodontic tooth movement, but its knockout increases osteoclastogenesis, resulting in faster tooth movement.

lncRNAs respond to orthodontic force as mechano-responsive regulators and serve as guides, signals, decoys, or scaffolds, controlling transcription and epigenetic programs in the PDL, and they influence osteoblast–osteoclast coupling [70]. Malocclusion develops when the modeling or remodeling of the jaws or alveolus is imbalanced; therefore, lncRNAs that regulate these processes under force are functionally linked to growth modification and the capacity for orthodontic correction. Disrupted alveolar bone turnover and tooth developmental anomalies underpin many malocclusions. lncRNAs that regulate these pathways contribute to skeletal growth patterns and susceptibility to orthodontic displacement [71]. Additionally, the ZFAS1 lncRNA may influence tooth development and alveolar bone resorption. Disruptions in alveolar bone turnover and anomalies in tooth development contribute to many malocclusions. lncRNAs that control these pathways affect skeletal growth patterns and susceptibility to orthodontic displacement.

Circular RNA is not often studied as miRNA, although a single circRNA can inactivate many miRNAs. A 2024 review on circRNAs in oral tissue-derived stem cells reports that circRNAs (1) regulate osteogenic and odontogenic differentiation of PDL stem cells (PDLSCs) and dental pulp stem cells (DPSCs); act as miRNA sponges, influencing key signaling pathways involved in alveolar bone remodeling; and (3) participate in the mechanical induction of alveolar bone remodeling, which is the fundamental biological process behind orthodontic tooth movement [72]. Because malocclusion arises from dysregulated craniofacial growth and abnormal alveolar bone remodeling, these findings suggest that circRNAs could influence malocclusion by regulating bone modeling and remodeling. Another study reports circRNA-mediated regulation of PDLSCs, suggesting its role in orthodontic force adaptation [73]. This study shows that circRNA CDR1as regulates PDLSC proliferation under inflammatory conditions and acts as an miR-7 sponge, thereby modulating extracellular signal-regulated kinase (ERK) signaling and influencing PDL cell behavior. Given that PDL biology directly governs tooth movement and tissue adaptation, circRNAs involved in PDL homeostasis may shape individual variability in malocclusion development or orthodontic treatment response. This evidence supports the idea that circRNAs embedded in PDLs likely play underexplored roles in the biomechanics of malocclusion. Therefore, although direct evidence linking circRNAs to malocclusion is still emerging, multiple studies show that circRNAs regulate bone remodeling, PDL mechanotransduction, stem-cell differentiation, and inflammatory responses, all of which influence craniofacial growth and orthodontic adaptability. circRNAs are therefore credible molecular contributors to malocclusion and potential biomarkers for predicting orthodontic treatment outcomes.

As with other epigenetic mechanisms discussed in this review, it is important to distinguish evidence related to malocclusion etiology from evidence related to orthodontic tissue remodeling. A substantial proportion of current ncRNA research has been conducted in the context of orthodontic tooth movement and force-induced bone remodeling. Consequently, these studies provide strong mechanistic evidence for the role of ncRNAs in orthodontic treatment response but only indirect evidence for their involvement in the initial development of malocclusion.

### 4.4. RNA Modifications

Although evidence regarding the involvement of RNA modifications (epitranscriptomics) in dentistry is very limited, it should be recognized that such modifications are important for all aspects of gene expression, including genes encoding ncRNAs such as miRNAs. Therefore, the above results cited for the role of miRNAs in malocclusion can be related to RNA modifications. It was shown that epitranscriptomic controls mechanotransduction in PDL and alveolar bone [74].

At present, epigenetic RNA modifications are less extensively characterized, less mechanistically resolved, and less directly linked to stable phenotypic outcomes than DNA methylation and histone modifications, which have been studied for decades, while RNA epigenetic modifications have been intensively studied only in the past decade. Consequently, their more limited treatment reflects the current maturity of the field rather than conceptual neglect.

A 2025 study showed that m^6^A RNA methylation regulates skeletogenic pathways that shape craniofacial form [75]. This work demonstrated that (1) m^6^A RNA methylation regulates key pathways involved in skeletal development, including WNT (wingless-related integration site), TGF-β, BMP, and other signals crucial for facial bone growth; (2) m^6^A rewires osteoblast and osteoclast differentiation pathways, influencing bone modeling and balance; and (3) m^6^A writers (such as methyltransferase-like 3 (METTL3)/METTL14), erasers (like FTO, AlkB homolog 5, and ALKBH5), and readers (YTHDF family) control RNA stability and translation of growth-related genes. Improper maxillofacial bone shaping and remodeling can cause malocclusion; therefore, epitranscriptomic changes that affect these growth pathways may lead to abnormal jaw development or remodeling.

Studies showed that epitranscriptomic dysregulation is linked to developmental diseases affecting craniofacial structures [75,76]. Those studies demonstrated that RNA-modifying enzymes are involved in developmental disorders affecting skeletal and craniofacial tissues. m^6^A appears crucial for vertebrate skeletal patterning and may impact long-bone and craniofacial shape development. Malocclusions with skeletal components (such as retrognathia, prognathism, and vertical dysplasia) are partly developmental, so disruptions in epitranscriptomics can contribute to their causes.

In summary, although direct studies on epitranscriptomics and malocclusion are still emerging, there is convincing evidence that RNA modifications regulate craniofacial bone development, modulate bone remodeling and mechanosensing, and control ncRNA networks essential for orthodontic biology. Consequently, epitranscriptomic alterations represent a credible mechanistic contributor to both the development and orthodontic expression of malocclusion.

While the epigenetics of malocclusion highlights how aberrant craniofacial growth patterns and occlusal relationships can arise from environmentally modulated gene regulation during development, these mechanisms should not be regarded as static or irreversible. Epigenetic marks are inherently dynamic and responsive to biomechanical, inflammatory, and metabolic stimuli throughout life. In this context, malocclusion can be viewed not only as the phenotypic outcome of altered epigenetic programming but also as a condition embedded in a plastic regulatory landscape that can be further modified. This conceptual shift provides a biological framework for understanding how mechanical interventions applied during orthodontic treatment may interact with and potentially reshape epigenetic regulation in periodontal, alveolar bone, and dental tissues.

Orthodontic tooth movement represents a unique clinical model of controlled mechanical loading that induces highly coordinated cellular and molecular responses, including sterile inflammation, bone remodeling, and ECM reorganization. Emerging evidence suggests that these processes are accompanied by treatment-induced epigenetic modifications that modulate the expression of genes involved in osteoclastogenesis, osteogenesis, angiogenesis, and mechanotransduction. Accordingly, orthodontic forces may act not only as physical drivers of tooth displacement but also as epigenetic stimuli that reprogram local cellular behavior, giving rise to the concept of epigenetically driven tooth movement. This perspective builds directly upon the epigenetic basis of malocclusion and positions orthodontic therapy as an active biological intervention capable of influencing gene–environment interactions, with potential implications for treatment efficiency, stability, and individual variability in response.

## 5. Epigenetic Modifications Induced by Orthodontic Treatment and the Concept of Epigenetically Driven Tooth Movement

Orthodontic tooth movement is primarily a biomechanical process in which controlled forces cause remodeling of the PDL and alveolar bone [77]. Increasing evidence indicates that this remodeling is accompanied by, and likely regulated through, rapid, dynamic epigenetic changes in cells exposed to mechanical stress [78]. These changes affect the transcription of genes vital for osteoclastogenesis, osteogenesis, inflammation, and extracellular matrix turnover. Some aspects of epigenetic links to OTM were discussed earlier, and now a more in-depth look into this issue will be provided. Although malocclusion is the main focus of this work, its treatment, which includes OTM, is also important.

### 5.1. Orthodontic Forces Modify Epigenetic Profiles

Mechanical compression and tension within the PDL are known to alter DNA methylation patterns in a force-dependent and time-dependent manner [79]. Reduced methylation (hypomethylation) at promoters of pro-resorptive genes, such as RANKL, MMPs, or inflammatory mediators, may facilitate osteoclast recruitment on the compression side [80]. Conversely, increased methylation (hypermethylation) at inhibitors of bone formation can support osteogenesis on the tension side. These methylation changes help explain individual differences in tooth movement rates and variability in treatment outcomes.

Orthodontic forces influence histone modifications, especially on histone H3 [81]. Increased acetylation correlates with gene activation in bone remodeling, while repressive marks such as H3K27me3 are altered during force application to regulate osteogenic and osteoclastic pathways [82]. These changes modify chromatin accessibility to transcription factors, enabling tissue adaptation to biomechanical stress.

Orthodontic tooth movement influences the expression of many miRNAs, lncRNAs, and circRNAs [70,83,84]. Certain miRNAs, elevated by compressive forces, promote RANKL expression and osteoclast activation [85]. Other miRNAs and lncRNAs support osteoblast differentiation when tensile forces are applied [86]. Non-coding RNAs function as rapid-response molecular switches, adjusting gene expression during the early and middle phases of tooth movement.

Although poorly explored in orthodontics, mechanical stress influences m6A RNA methylation in osteogenic tissues in other contexts [87]. It is plausible that RNA methylation dynamics take place in PDL cells during force application [88]. Therefore, m6A regulation may modify mRNA stability and translation of remodeling genes, providing a regulatory layer to force-induced adaptation.

### 5.2. Potential of Epigenetic Alteration to Induce Tooth Movement. A Conceptual Perspective

The concept of “epigenetically driven tooth movement” should currently be regarded as a theoretical framework intended to stimulate future investigation rather than as an evidence-supported biological mechanism. Although epigenetic pathways are known to regulate bone remodeling and orthodontic tooth movement, no experimental studies have demonstrated that targeted epigenetic manipulation alone can induce controlled and directional tooth movement in the absence of orthodontic force. The discussion below therefore explores a hypothesis derived from existing mechanistic observations and emerging biological principles.

Although there is no direct experimental evidence that targeted epigenetic modification alone can cause tooth movement without mechanical force, several lines of reasoning support the biological plausibility of this concept.

**Figure 3:**
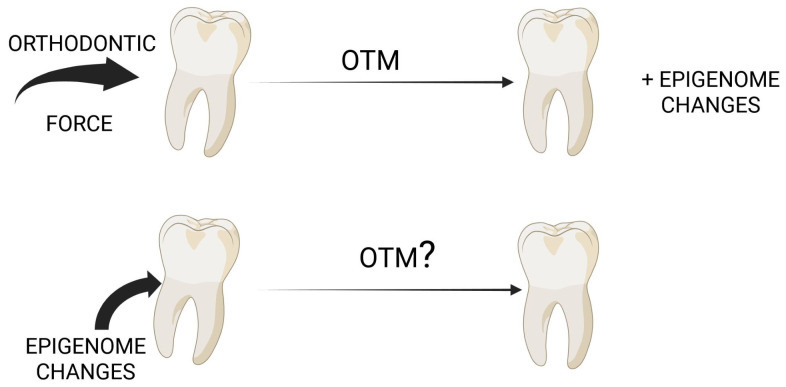
The “epigenetic tooth movement” concept. An orthodontic force applied to an abnormally positioned tooth corrects its position in the orthodontic tooth movement (OTM) and induces epigenetic changes in neighboring cells. The concept assumes that the same epigenetic changes might induce OTM. These changes are exemplified here as DNA demethylation of the receptor activator of nuclear factor κB ligand (RANKL) promoter, HDAC inhibition promoting osteoblast differentiation, and upregulation of mechanosensitive miRNAs. Created in BioRender. Michlewska, S. (2026) https://BioRender.com/asmghd5 (accessed on 2 July 2026).

Since tooth movement mainly depends on osteoclast-mediated resorption (on the compression side) and osteoblast-mediated formation (on the tension side), and both cell types are heavily regulated by epigenetic mechanisms, altering these pathways could, in theory, shift the remodeling balance enough to mimic the biological state caused by force [89] (Figure 3). Several effects support this idea, including the following: (1) demethylating RANKL promoters enhances RANKL expression by 170-fold and OPG expression by 20-fold [90]; (2) inhibiting HDAC activity promotes osteoblast differentiation [91]; and (3) upregulating mechanosensitive miRNAs creates a “pseudo-mechanical” remodeling signal [92]. However, without mechanical tension or compression to guide direction, remodeling would likely be uncoordinated or diffuse, resulting in uncontrolled tooth movement.

A more plausible alternative to force-free tooth movement is the idea that epigenetic intervention could enhance or accelerate OTM by increasing osteoclast recruitment, boosting bone turnover, improving the response to mechanical forces, and promoting faster resolution of inflammation [93]. Experimental models in bone biology indicate that inhibiting histone deacetylases or regulating miRNAs can significantly increase bone remodeling rates [94].

At present, the concept of epigenetically driven tooth movement should be viewed as a hypothesis-generating framework rather than a clinically applicable approach. Future experimental studies will be required to determine whether targeted modulation of epigenetic pathways can meaningfully enhance, direct, or partially replace conventional biomechanical stimulation.

If future technologies allow targeted delivery of epigenetic modulators to the PDL, such as HDAC inhibitors, miRNA mimics or inhibitors, lncRNA/circRNA modulators, m6A pathway modulators, or CRISPR/Cas9 epigenome editors, then biologically supported orthodontic therapy could become possible. These interventions might shorten treatment times, reduce the force needed, personalize treatment for slow responders, and mitigate age-related declines in remodeling ability. However, these interventions currently cannot produce controlled tooth movement without applying directional force, since directionality depends on biomechanical gradients.

## 6. Limitations of Current Evidence and Strength of Evidence

Despite growing interest in the role of epigenetics in craniofacial development and orthodontics, the current body of evidence remains limited in several key ways that constrain interpretation and clinical translation. First, there is a marked scarcity of direct human studies linking specific epigenetic modifications to clearly defined malocclusion phenotypes. Most available data come from in vitro systems or animal models, which, although mechanistically informative, do not fully capture the complexity of human craniofacial growth, genetic heterogeneity, and environmental exposure. As a result, causal inference remains limited. Second, the evidence is highly indirect. Many studies investigate general craniofacial development, bone biology, or OTM rather than malocclusion itself. While these findings provide important biological context, their direct applicability to clinical malocclusion is often inferential rather than demonstrative. Third, there is substantial heterogeneity in study design, model systems, and epigenetic endpoints. Differences in cell types, experimental conditions, methods of epigenetic analysis, and outcome measures limit comparability across studies and preclude quantitative synthesis. Fourth, most studies are cross-sectional or short-term, which restricts the ability to assess temporal relationships between epigenetic changes and craniofacial growth trajectories. The lack of longitudinal, phenotype-driven human studies represents a major barrier to establishing clinically relevant epigenetic markers. Fifth, although multiple epigenetic pathways have been implicated, there is a lack of standardized biomarkers and validated clinical endpoints, further limiting translational potential. Collectively, these limitations indicate that current evidence is better suited for generating hypotheses and mechanistic insights than for establishing clinically actionable conclusions.

To provide a structured overview, the available evidence was categorized by both its directness to malocclusion and its methodological strength (Table 1).

As summarized in Table 1, direct human evidence linking epigenetic alterations to specific malocclusion phenotypes remains scarce; therefore, much of the discussion necessarily relies on mechanistic and translational evidence from developmental, animal, and orthodontic models.

Overall, the most consistent evidence currently supports the involvement of epigenetic mechanisms in craniofacial development and orthodontic tissue remodeling, particularly through studies of bone remodeling, mechanotransduction, and orthodontic tooth movement. In contrast, direct evidence linking specific epigenetic alterations to clinically defined malocclusion phenotypes remains limited, and many proposed associations should be regarded as hypothesis-generating rather than confirmatory.

In summary, the current literature supports a biologically plausible role for epigenetic mechanisms in craniofacial development and orthodontic biology; however, the overall level of evidence is relevant but limited in confirmatory strength. Importantly, the absence of robust causal studies in humans and the predominance of indirect data warrant cautious interpretation. Consequently, epigenetic findings in malocclusion should currently be viewed as hypothesis-generating rather than definitive, and their translation into clinical orthodontic practice remains a perspective.

## 7. Perspectives and Outstanding Questions

In light of the predominantly indirect and heterogeneous nature of current evidence, future research directions must prioritize studies that improve causal inference and clinical relevance. Epigenetic studies in orthodontics have grown significantly in recent years, demonstrating that DNA methylation, histone modifications, non-coding RNAs, and RNA modifications contribute to the dynamic regulation of craniofacial and dentoalveolar tissues [4]. Although our understanding is still developing, several major themes are clear from the available evidence.

Epigenetic mechanisms link mechanical forces to tissue remodeling [9]. Periodontal ligament fibroblasts, osteoblasts, osteoclast precursors, and condylar chondrocytes all undergo rapid epigenetic changes when subjected to orthodontic loading. These changes reprogram gene expression patterns that control osteoclast recruitment, osteoblast differentiation, inflammatory signaling, and extracellular matrix remodeling. This mechanosensitive epigenetic regulation helps clarify the predictable biological stages of orthodontic tooth movement and explains individual differences in response to force.

Epigenetic variation may lead to differences among individuals in growth patterns, malocclusion risk, and treatment outcomes [6]. Patients with similar clinical traits often vary significantly in how quickly their teeth move, the stability of anchorage, and the risk of relapse. This variability might be due to inherited epigenetic states, early developmental programming, environmental influences, or accumulated life-course factors. Understanding these elements could help improve the timing of growth-modification methods, refine the application of force, and develop targeted strategies for “slow responders.”

Epigenetic mechanisms link environmental factors to craniofacial development, providing a biological basis for how airway obstruction, muscular activity, dietary consistency, endocrine disruptors, systemic health, and metabolic status influence malocclusion [4]. These findings highlight the importance of a holistic, personalized approach to orthodontic diagnosis and treatment planning.

Despite growing evidence from animal models and in vitro systems implicating epigenetic mechanisms in craniofacial development and malocclusion, a substantial translational gap remains when extrapolating these findings to humans. Experimental models enable precise control of genetic background, mechanical loading, and environmental variables, allowing causal links between specific epigenetic modifications and craniofacial phenotypes to be established. In contrast, human malocclusion arises from a complex interplay of genetic heterogeneity, developmental timing, functional habits, and environmental exposures, making it difficult to isolate epigenetic effects from confounding influences. Moreover, access to relevant human craniofacial tissues is limited, and most existing human studies rely on peripheral samples or cross-sectional designs that cannot capture spatiotemporal epigenetic dynamics during growth. As a result, while animal and in vitro studies provide critical mechanistic insights, their translation to human malocclusion remains inferential, underscoring the need for longitudinal, tissue-specific, and integrative clinical studies to validate epigenetic signatures with functional and clinical relevance.

The emerging field of epigenome-targeting technologies, including CRISPR-based epigenetic editing, ncRNA therapies, and local delivery of epigenetic modulators, holds significant promise. While clinical use is not yet practical, initial evidence indicates that biological modulation of remodeling pathways could accelerate OTM, stimulate bone formation, improve anchorage control, and reduce lag phases and hyalinization.

Epigenetic mechanisms constitute a critical, previously underrecognized dimension of craniofacial biology and orthodontic biomechanics. They regulate how tissues sense and respond to mechanical forces, integrate environmental signals, and determine growth trajectories and remodeling potential. Although many questions remain unanswered, epigenetic insights already enrich our understanding of dentofacial development and lay the foundation for more personalized, biologically informed orthodontic care.

Future progress will depend on multidimensional research approaches that combine molecular epigenetics, advanced imaging, biomechanics, and clinical data. Ultimately, the integration of epigenetic knowledge into orthodontics may lead to novel diagnostic tools, predictors of treatment response, biologically assisted therapies, and improved long-term stability.

Several emerging directions directly related to malocclusion and its treatment hold particular promise: (1) In personalized orthodontics guided by epigenetic profiling, epigenetic biomarkers can help predict individual rates of tooth movement, identify the best treatment timing, or determine susceptibility to skeletal discrepancies. (2) Targeted modulation of DNA methylation, histone acetylation, or ncRNA pathways may accelerate OTM or decrease the force required, increasing patient comfort and treatment efficiency. (3) Epigenetic insights into growth centers, including the mandibular condyle and sutures, may improve approaches to skeletal modification, especially during growth spurts. (4) Understanding how environmental exposures influence craniofacial epigenomes could lead to early preventive strategies in at-risk children. (5) Therapeutics that reset or stabilize epigenetic states in PDL and alveolar bone might reduce relapse and shorten retention periods. A major priority for future research is validating currently proposed epigenetic mechanisms in longitudinal human studies with clearly defined malocclusion phenotypes.

Some outstanding questions should be addressed in future research. Which specific epigenetic signatures distinguish skeletal malocclusion phenotypes? To what extent do early-life environmental exposures produce lasting epigenetic effects relevant to malocclusion? How stable are force-induced epigenetic changes, and can they be modulated to improve treatment outcomes or reduce relapses? Can epigenetic biomarkers reliably predict individual responses to orthodontic or orthopedic forces? Are RNA modifications, such as m6A, key regulators of bone remodeling in the PDL and alveolar bone? Could targeted epigenome editing, like CRISPR/Cas9, be safely used on dentoalveolar tissues? What is the relationship between systemic diseases, their epigenetic signatures, and malocclusion biology? How does aging affect the epigenome of craniofacial tissues, and what are the implications for adult orthodontics?

Overall, epigenetics is a promising avenue for research on the pathogenesis and treatment of malocclusion, but additional studies, as outlined above, are necessary to establish a mechanistic link between epigenomic changes and the clinical presentation of this disease. This would be confirmed through direct experimental and clinical research and individual variability in response.

## 8. Conclusions

The present review highlights that epigenetic mechanisms constitute a biologically plausible and increasingly studied dimension of craniofacial development and orthodontic biology. DNA methylation, histone modifications, and non-coding RNAs are supported by varying degrees of experimental evidence, whereas RNA modifications represent an emerging area that requires substantial further validation regarding their involvement in regulating osteogenesis, chondrogenesis, mechanotransduction, and tissue remodeling processes relevant to malocclusion and OTM.

However, consistent with the structured appraisal presented in this work, current evidence remains predominantly indirect and heterogeneous, with a strong reliance on in vitro systems, animal models, and studies of general craniofacial biology rather than on investigations that directly address clinically defined malocclusion phenotypes in humans. Importantly, robust causal evidence linking specific epigenetic modifications to specific types of malocclusion is currently lacking, and the overall strength of evidence must therefore be interpreted as moderately relevant but with limited confirmatory power.

Consequently, although epigenetic mechanisms offer a compelling framework for understanding gene–environment interactions in craniofacial development, their clinical applicability in orthodontics remains limited. At present, epigenetic findings should be considered hypothesis-generating rather than clinically actionable, and their use for diagnosis, treatment planning, or prediction of therapeutic outcomes has not yet been validated.

The strongest evidence currently concerns the role of epigenetic mechanisms in craniofacial biology and orthodontic tissue remodeling. By contrast, evidence supporting a direct etiological role of specific epigenetic alterations in defined malocclusion phenotypes remains comparatively limited and requires validation in human studies.

Future progress in this field will depend on the development of well-designed, longitudinal human studies that integrate precise phenotypic classification of malocclusion with tissue-specific and temporally resolved epigenetic profiling. Such approaches are essential for establishing causal relationships, identifying reliable biomarkers, and bridging the gap between biological plausibility and clinical utility.

In summary, epigenetics offers an important conceptual extension to genetic models of malocclusion; however, substantial methodological and translational challenges must be addressed before these insights can be effectively translated into clinical orthodontic practice.

## Figures and Tables

**Table 1:** Strength of evidence linking epigenetic mechanisms and malocclusion.

Mechanism/Study	Evidence	Directness	Strength	Key Limitations
DNA methylation	Mainly in vitro and animal, limited human data	Indirect	Moderate	Few phenotype-specific studies; mainly mechanistic research
Histone modifications	Animal and in vitro	Indirect	Moderate/low	Lack of human data; limited functional validation
ncRNA	In vitro, OTM *^a^* models, some human tissues	Semi-direct	Moderate	Mostly related to OTM, not malocclusion per se
RNA modifications (Epitranscriptomics)	Emerging	Indirect	Not determined	Very limited data; early-stage field
Mechanotransduction-related epigenetics	In vitro and animal OTM models	Semi-direct	Moderate	Focus on treatment response rather than etiology
Direct epigenetic studies in malocclusion patients	Sparse human studies	Direct	Low	Small sample sizes; lack of replication

*^a^*—orthodontic tooth movement.

## Data Availability

No new data were created or analyzed in this study. Data sharing is not applicable to this article.
